# A recurrence-free survivor with chemotherapy-refractory small cell lung cancer after pneumonectomy: A case report and review of the literature

**DOI:** 10.1097/MD.0000000000008922

**Published:** 2017-11-27

**Authors:** Yong Pan, Feng-Wei Kong, Heng Wang, Xiang Wang, Hui Zhang, Wen-Bin Wu, Miao Zhang

**Affiliations:** a Department of General Surgery, Xuzhou Infectious Disease Hospital, Xuzhou, China; b Department of Thoracic Surgery; c Department of Oncology, Xuzhou Central Hospital Affiliated to Southeast University, Xuzhou, China.

**Keywords:** apatinib, salvage surgery, small cell lung cancer (SCLC), targeted therapy

## Abstract

**Rationale:**

The optimal therapeutic regimen for chemotherapy-refractory and node-positive small-cell lung cancer (SCLC) is criticizable for the lack of evidence.

**Patient concerns:**

A patient with locally advanced SCLC was insensitive to the first-line chemotherapy of etoposide, irinotecan, and cisplatin.

**Diagnoses:**

The patient was diagnosed as SCLC with mediastinal lymph node metastasis by pathological staining.

**Interventions:**

Salvage pneumonectomy and systematic lymph node dissection combined with oral apatinib and mediastinal radiotherapy were performed for him.

**Outcomes:**

The patient survived for more than 2 years without recurrence after the operation and adjuvant therapy.

**Lessons:**

For patients with chemotherapy-resistant but resectable SCLC, a timely resection combined with postoperative radiotherapy and apatinib might be effective.

## 1 Introduction

Small cell lung cancer (SCLC) is characterized by rapid metastasis and widespread dissemination. The therapeutic regimen of SCLC has not changed significantly in the past several decades. Although it is sensitive to initial chemo-radiotherapy, long-term survivors are rare. The 5-year survival rate of these patients remains low (<7%), and most patients survive only for 1 year or less after diagnosis.^[1]^ Surgery (lobectomy and systemic lymph node dissection) could only be considered for strictly selected patients with cT1-2N0M0 SCLC,^[2,3]^ which is associated with improved long-term survival as compared to concurrent chemoradiotherapy. To date, no definitive regimen has been established for patients with chemotherapy-refractory SCLC. The role of aggressive surgery and targeted therapy has not been approved for the lack of evidence, despite the poor efficacy of the other treatment options. Meanwhile, the patients would take the risk of accelerated metastasis and worse prognosis of this devastating disease after surgery. However, it is reported that surgery may be actually underused for patients with early stage SCLC.^[4]^ Adjuvant radiotherapy followed by prophylactic cranial irradiation (PCI) might be effective for node-positive patients. There are still no approved targeted drugs for SCLC. Apatinib, an antiangiogenesis agent targeting vascular endothelial growth factor receptor-2 (VEGFR-2), demonstrates satisfactory efficacy in advanced nonsmall cell lung cancer (NSCLC) patients.^[5]^

Herein, a locally advanced SCLC patient who was resistant to the first-line chemotherapy was presented for discussion. To the best of our knowledge, this is the first report of salvage surgery combined with apatinib for stage IIIA SCLC, who gained recurrence-free survival for more than 2 years after surgery.

## 2 Case presentation

**Figure 1:**
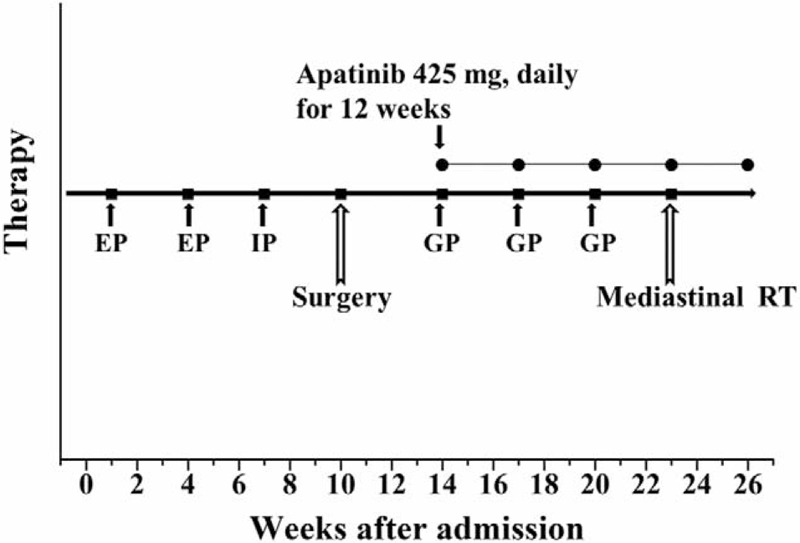
(A) CT scan on June 5, 2015 showed progressed central-type lung cancer after the first cycle chemotherapy using etoposide. (B) The tumor enlarged after the second cycle of etoposide. (C) The tumor progressed after the third cycle of chemotherapy using irinotecan. (D) Pathological staining of a tumor section indicated SCLC, by H-E staining (×200), on August 7, 2015. CT = computed tomography, SCLC = small-cell lung cancer.

A 54-year-old, male coal miner without smoking or drinking history was admitted to our hospital on May 9, 2015. His major complaint was discontinuous irritating cough, without hemoptysis, fever, or significant loss of weight. His family history indicated nothing abnormal. Physical examination showed respiratory harshness, without palpable supraclavicular lymph nodes. Related examinations were carried out step by step for differential diagnosis, such as pneumonia, tuberculosis, silicosis, and lung cancer. The laboratory tests including white blood cell count, cytokeratin 19 fragment (CYFRA 21-1), squamous cell carcinoma (SCC), neuron specific enolase (NSE), and carcinoembryonic antigen (CEA) were in normal range. His chest computed tomography (CT) revealed a central-type pulmonary mass measuring 2.0 cm × 2.0 cm in size and enlarged mediastinal lymph nodes, which involved the right middle and lower lobes (Fig. 1A). The tumor invaded the main bronchus but > 2 cm away from the carina. Fine-needle biopsy under bronchoscopy revealed the pathological diagnosis of SCLC. Biopsy of the lymph nodes by endobronchial ultrasound for staging was avoided, with the aim to diminish iatrogenic tumor dissemination.

Then the patient was clinically staged as IIIA (cT2N2M0), and he was not a candidate for surgery according to the updated guideline.^[3]^ But the patient showed progressive disease (PD) after two cycles of chemotherapy using etoposide (100 mg/day, for 4 days) and cisplatin (75 mg/m^2^ of body surface area) (EP), followed by one cycle of irinotecan (60 mg/m^2^ of body surface area, on day 1 and day 8) and cisplatin (75 mg/m^2^ of body surface area) (IP), as indicated morphologically in CT (Fig. 1B and C). Although peripheral pneumonia could not be excluded, the patient showed normal temperature without productive cough. Based on the above findings, he was considered to be chemotherapy refractory. After a second multidisciplinary consultation by thoracic surgeons and oncologists, salvage surgery combined with oral apatinib was considered to be the only choice for this rapidly aggravated disease. The patient probably loses the chance of single-stage radical resection after another neoadjuvant therapy. It was approved by Ethical Committee of our hospital.

Further abdomen CT, enhanced cranial magnetic resonance image (MRI), and bone emission computed tomography (ECT) excluded distant metastasis. Positron emission tomography was not carried out, because it was not covered by his health insurance. After his informed consent, pneumonectomy with mediastinal lymph node dissection was performed on August 7, 2015, strictly in accordance with the principles of oncological surgery.

The postoperative recovery was mainly uneventful, and the patient was discharged 16 days after surgery. Postoperative pathological staining of the specimen confirmed the diagnosis of SCLC (Fig. 1D), with positive expression of thyroid transcription factor 1, synaptophysin, chromogranin A and neural cell adhesion molecule. Besides, the lymph nodes in stations of 4, 7, and 10 were tumor involved. The resection margin was tumor free. Then the patient was pathologically staged as IIIA (pT2N2M0).

**Figure 2:**
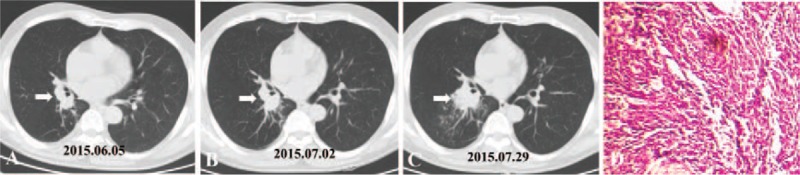
The schematic illustration of therapeutic regimen of the patient. EP = etoposide and cisplatin, GP = gemcitabine and cisplatin, IP = irinotecan and cisplatin, RT = radiotherapy.

One month after the surgery, adjuvant chemoradiotherapy and targeted therapy were carried out simultaneously. His therapeutic regimen was illustrated in Fig. 2. First, oral apatinib 425 mg once daily was administered for 3 months, and then it was discontinued because of persistent moderate anemia, fatigue, hand-foot syndrome, oral ulcerative mucositis, and hypertension. Three cycles of second-line chemotherapy using gemcitabine (1000 mg/m^2^ over 30 minutes, on day 1 and day 8, every 21 days) and cisplatin (75 mg/m^2^, every 21 days) (GP) were administered. Subsequently, the patient received mediastinal radiotherapy with a total dose of 50 Gy (2 Gy per day and 5 days/week), and 10 mg of nedaplatin on the first day was given as a radiosensitizer.

The patient refused prophylactic cranial irradiation (PCI) because of his compromised quality of life. During the follow-up, his serum tumor markers of CYFRA 21–1, SCC, NSE, and CEA were in normal range. He survived without local recurrence or distant metastasis for more than 2 years after the surgery up to now.

## 3 Discussion

The median survival time of patients with extensive stage SCLC is 9.4 to 12.8 months, and the 2-year survival rate is 5.2% to 19.5%.^[6]^ Resistance-to-conventional therapy and high-recurrence rate are ascribed to the heterogeneous genetic structure of SCLC.^[7]^ Benefit from second-line chemotherapy in SCLC patients is limited. Moreover, treatment for SCLC patients who are resistant to platinum-based chemotherapy is not yet elucidated. New therapeutic targets have emerged, but no significant improvement has been demonstrated thus far for SCLC.^[8]^ Due to the dismal prognosis of SCLC, novel effective treatments are urgently needed. As for this case, several issues might contribute to his long-term, recurrence-free survival.

First, many studies on antiangiogenesis agents for SCLC are ongoing.^[9]^ However, the role of angiogenesis inhibitors for SCLC is controversial. A meta-analysis indicates that adding angiogenesis inhibitors to chemotherapy does not improve the progression-free survival (PFS), overall survival, objective response rate, or 1-year PFS of SCLC patients.^[10]^ Besides, maintenance with targeted therapy fails to improve the survival of patients with limited or extensive-stage SCLC.^[11]^ Specifically, 7.5 mg/kg bevacizumab after induction chemotherapy does not improve the oncological outcomes in extensive-stage SCLC patients.^[12]^ The addition of rilotumumab or ganitumab to etoposide and carboplatin/cisplatin does not indicate improved benefit for extensive-stage SCLC.^[13]^ Comprehensive genomic analysis of SCLC might be a choice to identify patients who could benefit from targeted therapy.^[14,15]^

Second, in addition to concurrent chemoradiotherapy, aggressive surgery is beneficial to potentially curable limited-stage SCLC.^[16]^ Besides, an accurate staging is critical before surgery. Multidisciplinary therapies make more patients eligible for surgical resection. The assessment of circulating tumor cells in SCLC patients seems to be a precise method to detect tumor dissemination, which is potentially helpful in selection of patients who are suitable for surgery.^[17]^ However, the evidence from currently available randomized controlled trials does not support surgical resection for limited-stage SCLC as compared with chemoradiotherapy alone.^[18]^ Indications of salvage surgery include locally progressive and recurrent tumors,^[19]^ nevertheless, it might be technically more difficult with higher morbidity.^[20]^

It is noteworthy that the standard therapy for locally advanced chemotherapy-refractory SCLC is radiotherapy. Gemcitabine could be recommended for patients who have relapsed in 6 months after primary therapy,^[3]^ which has modest activity in previously treated SCLC patients.^[21]^ Pneumonectomy should not be recommended as first treatment. Besides, after refractory to platinum-based chemotherapy, cisplatin, or nedaplatin should not be recommended in the third line treatment. Apatinib has not been approved as second- or third-line therapy for SCLC in the NCCN guideline. As for this presented case, radical resection of the tumor and postoperative radiotherapy might affect more than oral apatinib on the recurrence-free survival of this patient, but the role of apatinib in SCLC worth further research.

## 4 Conclusion

Timely salvage surgery combined with radiotherapy and apatinib might be effective for patients with chemotherapy-refractory SCLC. However, high-quality studies regarding the exact efficacy of antiangiogenic agent and surgery are necessary.

## References

[1] Byers LA Rudin CM Small cell lung cancer: where do we go from here? Cancer 2015;121:664–72. 25336398 10.1002/cncr.29098 PMC5497465

[2] Rudin CM Ismaila N Hann CL Treatment of small-cell lung cancer: American Society of Clinical Oncology Endorsement of the American College of Chest Physicians Guideline. J Clin Oncol 2015;33:4106–11. 26351333 10.1200/JCO.2015.63.7918

[3] National Comprehensive Cancer Network. NCCN Clinical Practice Guidelines in Oncology: small cell lung cancer, Version 3.2017. Available at: https://www.nccn.org/professionals/physician_gls/pdf/sclc.pdf. Accessed May 23, 2017.

[4] Yang CJ Chan DY Shah SA Long-term survival after surgery compared with concurrent chemoradiation for node-negative small cell lung cancer. Ann Surg 2017;doi: 10.1097/SLA.0000000000002287. 10.1097/SLA.0000000000002287 28475559

[5] Ding L Li QJ You KY The use of apatinib in treating nonsmall-cell lung cancer: case report and review of literature. Medicine (Baltimore) 2016;95:e3598. 27196461 10.1097/MD.0000000000003598 PMC4902403

[6] Asai N Ohkuni Y Kaneko N Relapsed small cell lung cancer: treatment options and latest developments. Ther Adv Med Oncol 2014;6:69–82. 24587832 10.1177/1758834013517413 PMC3932054

[7] Kahnert K Kauffmann-Guerrero D Huber RM SCLC-state of the art and what does the future have in store? Clin Lung Cancer 2016;17:325–33. 27397481 10.1016/j.cllc.2016.05.014

[8] Koinis F Kotsakis A Georgoulias V Small cell lung cancer (SCLC): no treatment advances in recent years. Transl Lung Cancer Res 2016;5:39–50. 26958492 10.3978/j.issn.2218-6751.2016.01.03 PMC4758968

[9] Schneider BJ Kalemkerian GP Personalized therapy of small cell lung cancer. Adv Exp Med Biol 2016;890:149–74. 26703804 10.1007/978-3-319-24932-2_9

[10] Li Q Wu T Jing L Angiogenesis inhibitors for the treatment of small cell lung cancer (SCLC): a meta-analysis of 7 randomized controlled trials. Medicine (Baltimore) 2017;96:e6412. 28353568 10.1097/MD.0000000000006412 PMC5380252

[11] Roviello G Zanotti L Cappelletti MR No advantage in survival with targeted therapies as maintenance in patients with limited and extensive-stage small cell lung cancer: a literature-based meta-analysis of randomized trials. Clin Lung Cancer 2016;17:334–40. 27346522 10.1016/j.cllc.2016.05.008

[12] Pujol JL Lavole A Quoix E Randomized phase II-III study of bevacizumab in combination with chemotherapy in previously untreated extensive small-cell lung cancer: results from the IFCT-0802 trial†. Ann Oncol 2015;26:908–14. 25688059 10.1093/annonc/mdv065

[13] Glisson B Besse B Dols MC A randomized, placebo-controlled, phase 1b/2 study of rilotumumab or ganitumab in combination with platinum-based chemotherapy as first-line treatment for extensive-stage small-cell lung cancer. Clin Lung Cancer 2017;18:615.e8–25.e8. 28601388 10.1016/j.cllc.2017.05.007

[14] Umemura S Tsuchihara K Goto K Genomic profiling of small-cell lung cancer: the era of targeted therapies. Jpn J Clin Oncol 2015;45:513–9. 25670763 10.1093/jjco/hyv017

[15] Santarpia M Daffina MG Karachaliou N Targeted drugs in small-cell lung cancer. Transl Lung Cancer Res 2016;5:51–70. 26958493 10.3978/j.issn.2218-6751.2016.01.12 PMC4758978

[16] Almquist D Mosalpuria K Ganti AK Multimodality therapy for limited-stage small-cell lung cancer. J Oncol Pract 2016;12:111–7. 26869647 10.1200/JOP.2015.009068

[17] Hamilton G Rath B Ulsperger E A review of the role of surgery for small cell lung cancer and the potential prognostic value of enumeration of circulating tumor cells. Eur J Surg Oncol 2016;42:1296–302. 27402116 10.1016/j.ejso.2016.04.063

[18] Barnes H See K Barnett S Surgery for limited-stage small-cell lung cancer. Cochrane Database Syst Rev 2017;4:CD011917. 28429473 10.1002/14651858.CD011917.pub2 PMC6478097

[19] Van Breussegem A Hendriks JM Lauwers P Salvage surgery after high-dose radiotherapy. J Thorac Dis 2017;9 (suppl 3):S193–200. 28446984 10.21037/jtd.2017.03.88 PMC5392543

[20] Uramoto H Current topics on salvage thoracic surgery in patients with primary lung cancer. Ann Thorac Cardiovasc Surg 2016;22:65–8. 26948299 10.5761/atcs.ra.16-00019 PMC4936213

[21] Masters GA Declerck L Blanke C Phase II trial of gemcitabine in refractory or relapsed small-cell lung cancer: Eastern Cooperative Oncology Group Trial 1597. J Clin Oncol 2003;21:1550–5. 12697880 10.1200/JCO.2003.09.130

